# Multiomics Analysis Reveals Cuproptosis-Related Signature for Evaluating Prognosis and Immunotherapy Efficacy in Colorectal Cancer

**DOI:** 10.3390/cancers15020387

**Published:** 2023-01-06

**Authors:** Rong He, Heping Zhang, Huaxin Zhao, Xiaolan Yin, Jingyi Lu, Cheng Gu, Jie Gao, Qing Xu

**Affiliations:** Department of Oncology, Shanghai Tenth People’s Hospital, School of Medicine, Tongji University, Shanghai 200072, China

**Keywords:** cuproptosis, colorectal cancer, prognostic signature, immunotherapy, single-cell analysis

## Abstract

**Simple Summary:**

Cuproptosis is a newly discovered copper-dependent cell death. We aimed to explore the functions of cuproptosis in the tumor microenvironment and construct a cuproptosis-related prognosis signature for survival prediction and immunotherapeutic strategies. We comprehensively analyzed single-cell RNA-seq and bulk RNA-seq data from multiple colorectal cancer cohorts based on TCGA and GEO databases in the current study. The relationship between molecular clusters, clinical outcomes, and immune cell infiltration characteristics associated with cuproptosis was investigated. Considering the heterogeneity of colorectal cancer development, we then established a validated five-gene panel for predicting individual patient prognosis, drug sensitivity, tumor-immune microenvironment, and immunotherapy targets.

**Abstract:**

Cuproptosis is a copper-induced form of mitochondrial cell death which is engaged in the proliferation and migration of a variety of tumors. Nevertheless, the role of cuproptosis in tumor microenvironment (TME) remodeling and antitumor therapy is still poorly understood. We characterized two diverse cuproptosis-associated molecular isoforms in CRC which exhibit distinct prognostic and TME characteristics. Subsequently, we constructed a cuproptosis-associated prognostic model containing five genes and divided the patients into a high CPS-score group and a low CPS-score group. Univariate and multivariate Cox analyses showed that the CPS score could be used as an independent prognostic factor. The nomogram, and its consequent calibration curves, indicated that this prognostic signature had good predictive power for CRC. The analysis of single-cell sequencing data showed the significant expression of HES4 and SPHK1 in various immune and stromal (including fibroblasts) cells. Further studies showed that tumor mutational burden (TMB), high microsatellite instability (MSI-H) ratio, immune checkpoint blockade (ICB), and human leukocyte antigen (HLA) gene expression all positively correlated with the CPS score, predicting a better reaction to immunotherapy in high CPS-core patients. The CPS score constructed from cuproptosis subtypes can be used as a predictive tool to evaluate the prognosis of CRC patients and their response to immunotherapy.

## 1. Introduction

Globally, colorectal cancer (CRC) is the third most frequent type of malignancy and the second leading cause of cancer-related deaths [1]. By 2020, approximately 1.9 million new cases of CRC were expected to occur and 90,000 people were expected to die, according to Global Cancer Statistics [2]. Compared to the past few decades, improvements in diagnosis, staging, and multimodal therapy have improved the local control and survival of patients with early-stage CRC [3]. Unfortunately, most patients with CRC are not diagnosed until late in the disease, resulting in complex surgery and an abysmal prognosis [4]. As a result of the extensive infiltration and distant metastases, the 5-year follow-up rates for advanced CRC cases are also incredibly low, at only 5–10% [5]. Recent studies have shown that multiple molecular marks, of which DNA mismatch repair (MMR) status is included, are available to distinguish CRC into distinct molecular subtypes, which has a remarkable impact on the treatment and outcome of CRC [6]. Defective DNA mismatch repair (d-MMR) is associated with tumor development and high microsatellite instability (MSI-H), which are observed in diverse kinds of carcinomas (such as colon and endometrial cancers) [7,8]. MSI-H/d-MMR tumors are characterized by high tumor mutation burden (TMB), lymphocytic infiltration, and the expression of immune checkpoints, which are thought to be associated with sensitivity to immune checkpoint inhibitors [9,10,11]. However, the MSI-H phenotype occurs in less than 15% of colorectal cancer patients, suggesting PD-1/PD-L1 immunotherapy will not benefit most CRC patients [12]. It is therefore imperative that methods be developed to improve the treatment of cancer patients with low microsatellite instability (MSI-L) or microsatellite stability (MSS) clinical response and prognosis.

Multicellular organisms have been found to undergo several types of programmed cell death (RCD), including apoptosis, pyroptosis, and necroptosis [13]. In a recent study, Tsvetkov et al. identified a new copper-dependent cell death mechanism, which they dubbed “cuproptosis” [14]. Intracellular copper (Cu) binds directly to the lipid acylation component of the tricarboxylic acid cycle, triggering the accumulation of lipid-acylated proteins and the reduction in Fe/S class proteins, which contributes to proteotoxic stress and, ultimately, to cuproptosis. It has been found that elevated levels of Cu in cells and tissues may facilitate the advancement of several neoplasm types [15,16]. For example, Cu has been shown to interact with MEK1 to promote MAPK signaling and tumor progression through oncogenic BRAF signaling precisely [17]. Furthermore, copper removal from the tumor microenvironment (TME) may damage Cu-dependent SOD enzymes and transform the M1 macrophages of the proinflammatory phenotype to the M2 macrophages of the procarcinoma phenotype and the formation of an immunosuppressive microenvironment [18]. Considering the critical role of Cu in the progression of carcinogenesis and TME, targeting Cu homeostasis will be an emerging anticancer therapeutic strategy. However, the associations of cuproptosis-associated regulators (CRs) with tumor characteristics, TME, and drug sensitivity in colorectal cancer patients remain unknown. Therefore, identifying cuproptosis-related gene signatures in colorectal cancer is essential to identify a novel diagnostic and therapeutic strategy.

In the current study, we comprehensively analyzed single-cell RNA-seq (scRNA-seq) and bulk RNA-seq data from multiple colorectal cancer cohorts. Based on CRs, we identified cuproptosis-associated molecular subtypes and linked CRs to clinical outcomes, gene mutations, and TME in CRC patients. Considering the heterogeneity of colorectal cancer progression, we built a 5-gene-based model that predicts the outcome of CRC patients, describes the immune microenvironment, and predicts the response of immunotherapy and sensitivity to antitumor drugs. In addition, the expression patterns of model genes were further inquired at the CRC single-cell level and in cell lines. In conclusion, this study established a new cuproptosis-related prognostic index (CPS score) for overall survival (OS) estimation in CRC patients and probed more accurate molecular phenotypes in CRC and the corresponding TME features.

## 2. Methods

### 2.1. Collection and Preprocessing of Transcriptomic Datasets

First, we captured gene transcriptome data, clinical features (*n* = 458), and mutation information (*n* = 452) from TCGA for normal and colorectal cancer samples. Fragments per kilobase were processed for transcriptomic data and translated to transcripts per million. Next, two colorectal cancer datasets with complete clinical information, including GSE17536 and GSE39582, were downloaded from GEO databases. The three datasets were combined using the “ComBat” algorithm and corrected for nonbiotechnologically-biased batch effects using the “SVA” R package [19]. In Supplementary Table S1, we present the clinical characteristics of all CRC patients.

### 2.2. Downloading and Processing of Single-Cell Sequencing Data

The CRC single-cell dataset GSE188711 was downloaded from the GEO database and contained six samples. Seurat objects were created using the “Seurat” R package, and high-quality cells were included in the subsequent analysis. We excluded cells with fewer than 200 genes and those with fewer than three genes detected, as well as cells with more than 5% mitochondrial genes. Log normalization of the combined data was carried out, and the 1500 genes with the highest intercellular coefficient of variation were then filtered using the “FindVariableFeatures” function. The t-distribution random neighbor-embedding (tSNE) algorithm was employed to dimensionally reduce scRNA-seq. In addition, the “FindAllMarkers” algorithm was executed to retrieve differentially expressed marker genes in separate cell clusters with a filter value of |log2 Fold Change (FC)| ≥ 1 and an adjusted *p*-value of <0.05. After that, cells were annotated with the “SingleR” package based on marker genes to determine cell subpopulations [20].

### 2.3. Unsupervised Clustering

Nineteen CRs were searched for in previous research and are listed in Supplementary Table S2 [14]. By utilizing the “limma” package, we identified differentially expressed CRs (DECRs) in colorectal cancer and normal tissues of the TCGA dataset with screening criteria of |Fold Change| ≥ 1.5 and false discovery rate (FDR) < 0.05. Unsupervised clustering analysis was performed on the meta-cohort of 1191 CRC patients based on DECRs to identify distinct cuproptosis-associated phenotypes. Our consensus clustering algorithm was performed using the “ConsensusClusterPlus” R package, with 1000 replications to ensure accuracy [21].

### 2.4. Enrichment Analysis and Differential Expression Analysis of Molecular Subtypes

The “GSVA” R package was utilized for enrichment analysis to identify variances in pathways among the distinct cuproptosis molecular phenotypes. The single-sample gene set enrichment analysis (ssGSEA) algorithm is employed for detecting the relative amount of specific immune cell infiltrations in individual CRC cases. Charoentong’s study provided gene sets for labeling immune cells infiltrating TME [22]. To identify differentially expressed genes (DEGs) between cuproptosis phenotypes, we used the “limma” package. The screening criteria were adjusted *p*-values < 0.05 and |Fold Change| ≥ 1.5. The “heatmap” package was used to visualize the expression of CRs in various cuproptosis phenotypes.

### 2.5. Derivation of Cuproptosis-Related Prognostic Signature

Based on survival information from the TCGA-CORD cohort, univariate Cox regression and multifactor Cox regression were used to establish the CPS score. GSE17536 and GSE39582 were used as the validation sets to confirm the predictive value of this signature. The optimal value of the score was calculated using the “survminer” R package, and patients were grouped into low CPS and high CPS-score subgroups. Kaplan–Meier (K-M) survival curves were used to compare patients’ overall survival (OS) times in the distinct CPS-score groups. Receiver operating characteristic (ROC) curves were used to assess the validity and accuracy of the model. By performing univariate and multivariate Cox regression analyses, we identified independent prognostic factors from clinical variables and the CPS score. A nomogram with clinical features was created using the “rms” R package to predict survival in CRC.

### 2.6. Assessment of Immune Microenvironmental Characteristics

To calculate the immune score and stromal score, we applied the “ESTIMATE” algorithm in R software to calculate TCGA-COAD data from each sample [23]. The “CIBERSORT” algorithm was used to assess the infiltration level of the twenty-two immune cell categories in CRC samples based on RNA-seq data [24]. Further, immune checkpoint blockade (ICB) and human leukocyte antigen (HLA) genes were assessed in distinct CPS-score subgroups.

### 2.7. Drug Sensitivity Analysis

Data from the CellMiner database were used to extract gene expression data and drug-sensitive information [25]. FDA-approved drugs were selected for analysis. The first 16 with strong correlations are displayed and sorted by the absolute value of the correlation.

### 2.8. Cell Culture and RT-qPCR

The normal adult intestinal cell line NCM460 and the human colorectal cancer cell lines HCT116, SW480, and SW620 were obtained from the Cell Bank of the Chinese Academy of Sciences (Shanghai, China). NCM460, HCT116, and SW480 were routinely cultured in Dulbecco’s modified Eagle’s medium (DMEM, Gibco, Fair Lawn, NJ, USA) containing 10% fetal bovine serum (FBS, Gibco, USA) and 1% antibiotics (penicillin/streptomycin); SW620 were routinely cultured in RPMI-1640 medium (Invitrogen Co., Waltham, MA, USA) containing 10% FBS and 1% antibiotics. The cells were grown at 37 °C with 5% CO_2_.

Total RNA was prepared from the cells using TRIzol reagent (Vazyme Biotech Co. Ltd., Nanjing, China) according to the manufacturer’s instructions. Subsequently, the extracted RNA was reverse transcribed using a HiScript II One-Step RT-PCR Kit (Vazyme Biotech Co. Ltd., Nanjing, China). A 2 × ChamQ Universal SYBR qPCR premix (Vazyme Biotech Co. Ltd., Nanjing, China) was used for all PCR reactions. The relative mRNA expression of genes was calculated using the 2^−ΔΔCt^ method and normalized to GAPDH. Primer sequences for the model genes are outlined in Supplementary Table S3.

### 2.9. Statistical Analysis

All of the statistical analyses were run with R software v4.2.1 (https://www.r-project.org/, accessed on 12 October 2022) and its associated packages. Statistical differences between groups were calculated via one-way ANOVA or student’s *t*-test with GraphPad Prism 8.0. Statistical significance was considered for two-tailed *p* < 0.05 unless otherwise stated.

## 3. Results

### 3.1. Expression and Mutational Landscape of CRs

**Figure 1:**
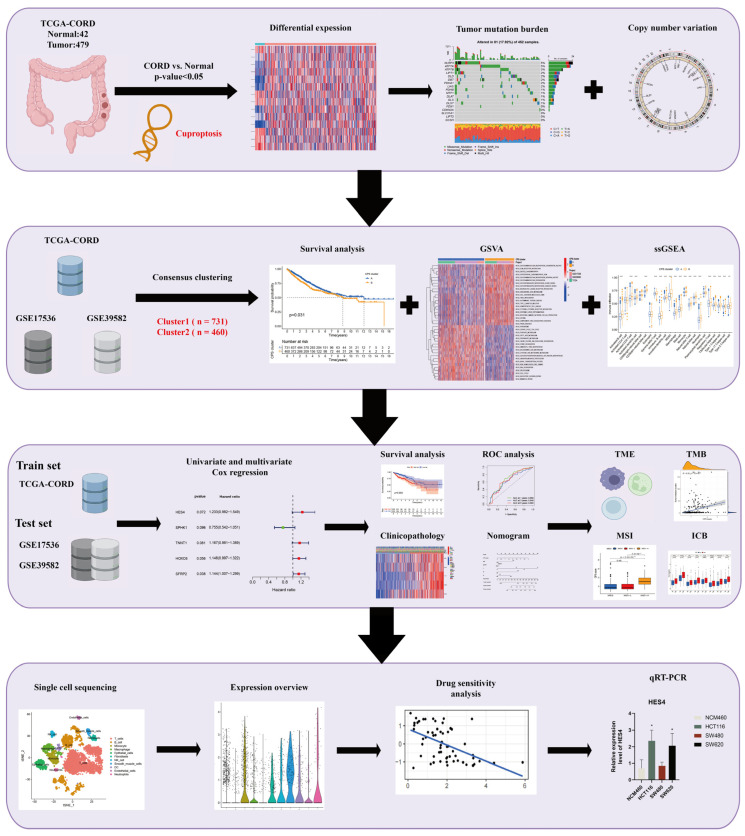
Flow chart of this study. * *p* < 0.05; ** *p* < 0.01; *** *p* < 0.001.

**Figure 2:**
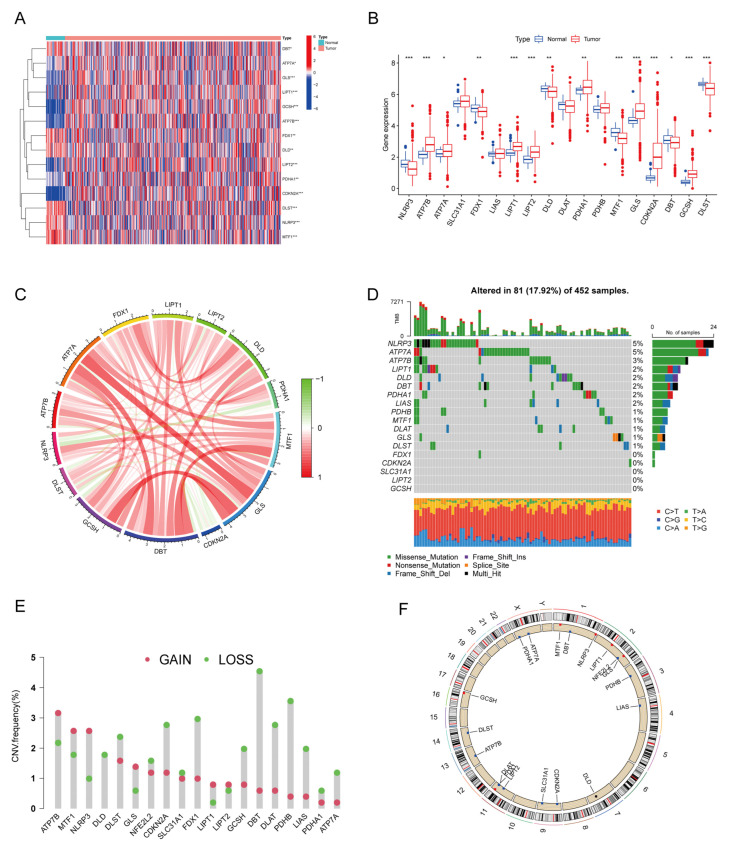
Expression levels and genetic variation in cuproptosis-associated regulators. Heatmap (**A**) and boxplot (**B**) of DECRs in colorectal cancer and normal tissues. (**C**) The association network shows the interactions between DECRs. Mutation frequencies (**D**) and copy number variant (**E**) of DECRs in colorectal cancer patients. (**F**) Location of DECRs on the chromosome. DECRs, differentially expressed cuproptosis-associated regulator, * *p* < 0.05; ** *p* < 0.01; *** *p* < 0.001.

The analysis workflow diagram for this study can be seen in Figure 1. To systematically pursue the potential role of CRs in CRC progression, we identified the expression levels of all 19 regulators in CRC and normal tissues. In total, 14 DECRs were identified (Figure 2A), of which six regulators (*MTF1*, *DLST*, *NLRP3*, *DBT*, *FDX1*, and *DLD*) were downregulated in cancer tissues (Figure 2B). In contrast, eight regulators (*PDHA1*, *ATP7A*, *LIPT1*, *LIPT2*, *GLS*, *ATP7B*, *GCSH*, and *CDKN2A*) were upregulated in cancer tissues. The correlation network of cuproptosis regulators was then plotted based on the expression level of each of the CRs to depict their intimate internal linkage (Figure 2C). Next, we further assessed genetic and transcriptomic variants in CRs. Regulatory mutations associated with cuproptosis were shown in 81 of 452 CRC samples (17.92%) and were mainly missense mutations (Figure 2D). Among these, *NLRP3* and *ATP7A* were the most frequently mutated genes (5%). We also observed that all CRs presented copy number variation (CNV) (Figure 2E). Most of them showed copy number deletions, with *DBT* and *PDHB* being the most pronounced. In contrast, *ATP7B*, *MTF1*, and *NLRP3* were dominated by copy number amplification. The chromosomal location of CNV mutations in CRs is indicated in the circle diagram (Figure 2F).

### 3.2. Identification of CRC Molecular Subtypes Based on Cuproptosis Regulators

**Figure 3:**
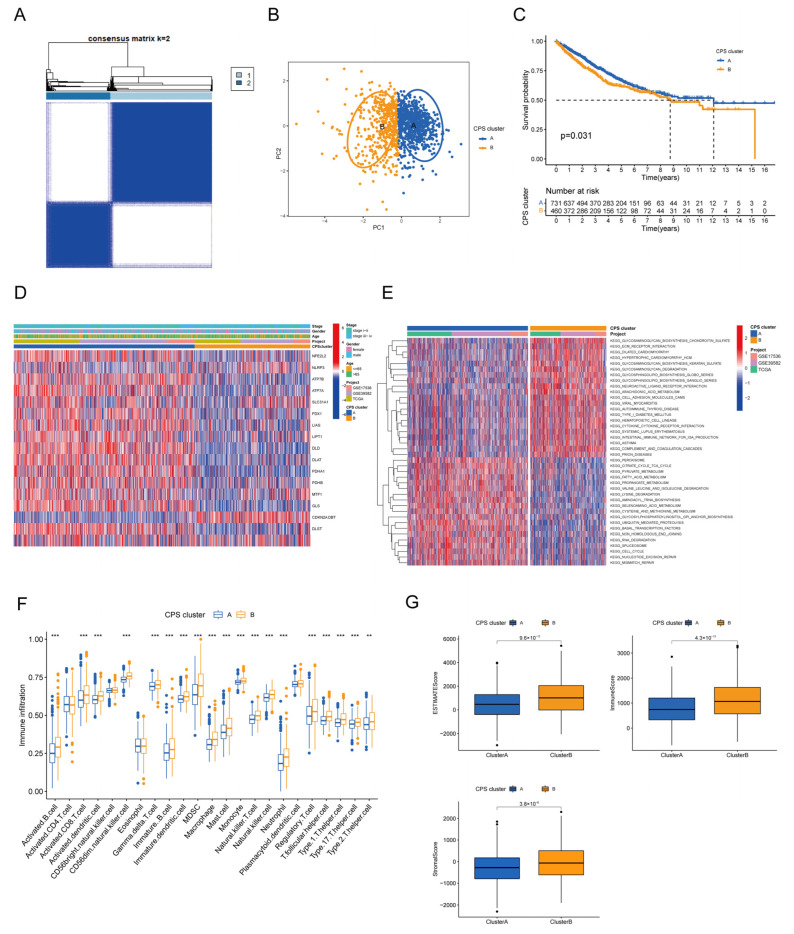
Identification of cuproptosis modification patterns in colorectal cancer by consensus clustering. (**A**) Two clusters were identified by consistent clustering analysis (k = 2). (**B**) Principal component analysis shows a different distribution between the two subtypes. (**C**) Kaplan–Meier survival curves of patients with different cuproptosis modification patterns. (**D**) Differences in clinicopathological characteristics and expression levels of CRs between the two cuproptosis subgroups. (**E**) Gene set variation enrichment analysis of patients with different cuproptosis modification patterns for ascertaining the activation status of biological pathways. (**F**) The abundance of immune cell infiltration between the different molecular subtypes. (**G**) ESTIMATE score, immune score, and stromal score between different molecular subtypes. CR, cuproptosis-associated regulator; ** *p* < 0.01; *** *p* < 0.001.

To fully understand the expression patterns and biology of cuproptosis regulators in CRC, we integrated three cohorts of patients with available survival information (TCGA cohort, GSE17536, and GSE39582), containing a total of 1191 CRC samples. Unsupervised clustering was used to analyze relevant subtypes in CRC, and we found the optimum clustering with a k value of 2. Patients were classified into two distinct clusters based on the expression of 14 DECRs, with CPS cluster-A comprising 731 cases and CPS cluster-B comprising 460 cases (Figure 3A). Principal component analysis (PCA) confirmed that the two clusters could be distinguished by the expression levels of 14 DECRs (Figure 3B). Survival analysis suggested that CRC patients in CPS cluster-A showed considerably better overall survival (OS) versus CPS cluster-B (*p* = 0.031, Figure 3C). Furthermore, we combined the molecular subtypes and clinicopathological features of CRC patients to create a clinically relevant heat map and found that most DECRs were highly expressed in CPS cluster-A (Figure 3D).

### 3.3. Biological Behavior and TME Characteristics of Distinct Cuproptosis Phenotypes

To elucidate the biological differences between different cuproptosis subtypes, we performed the KEGG pathway enrichment analysis with the GSVA algorithm. The GSVA details are presented in Supplementary Table S4. CPS cluster-A was significantly activated mainly in metabolic pathways such as the tricarboxylic acid cycle, pyruvate metabolism, and fatty acid metabolism. CPS cluster-B was significantly activated during glycosaminoglycan synthesis and degradation (chondroitin sulfate synthesis and glycosaminoglycan degradation) and matrix-related pathways such as ECM receptor interactions, cell adhesion, and cytokine receptor interactions (Figure 3E). Next, we sought to understand whether cuproptosis regulators had an effect on TME in CRC and thus performed ssGSEA enrichment analysis to characterize the immune cell infiltration landscape of different clusters. We observed multiple immune cell types, including activated B cells, activated CD8 T cells, activated dendritic cells, CD56dim natural killer cells, gamma delta T cells, immature B cells, immature dendritic cells, MDSC, macrophages, mast cells, monocytes, natural killer T cells, natural killer cells, neutrophils, regulatory T cells, T follicular helper cells which were significantly enriched in cluster-B (Figure 3F). Additionally, the ESTIMATE score, immune score, and stromal score of CPS cluster-B were significantly higher than those of CPS cluster-A (Figure 3G).

To further explore cuproptosis modification patterns in CRC, we identified 120 DEGs associated with the cuproptosis phenotype by using the limma package. All genes are shown in Supplementary Table S5. Consistent with cuproptosis phenotype clustering, this analysis classified patients into two distinct genomic subtypes, which we named gene cluster-A and gene cluster-B (Supplementary Figure S1A). Like CPS cluster-A, gene cluster-A had a significant survival advantage (*p* = 0.004, Supplementary Figure S1B). The expression of 15 cuproptosis regulators, except *LIAS*, *LIPT2*, *DBT*, and *GCSH*, differed significantly between the two gene manifestations (Supplementary Figure S1C). The heatmap demonstrated that CRC patients with distinct CPS clusters and gene clusters had dramatically diverse clinical traits and expression variations (Supplementary Figure S1D). These findings efficiently proved the striking validity of the DECRs-based clustering analysis.

### 3.4. Development and Verification of CPS Score

**Figure 4:**
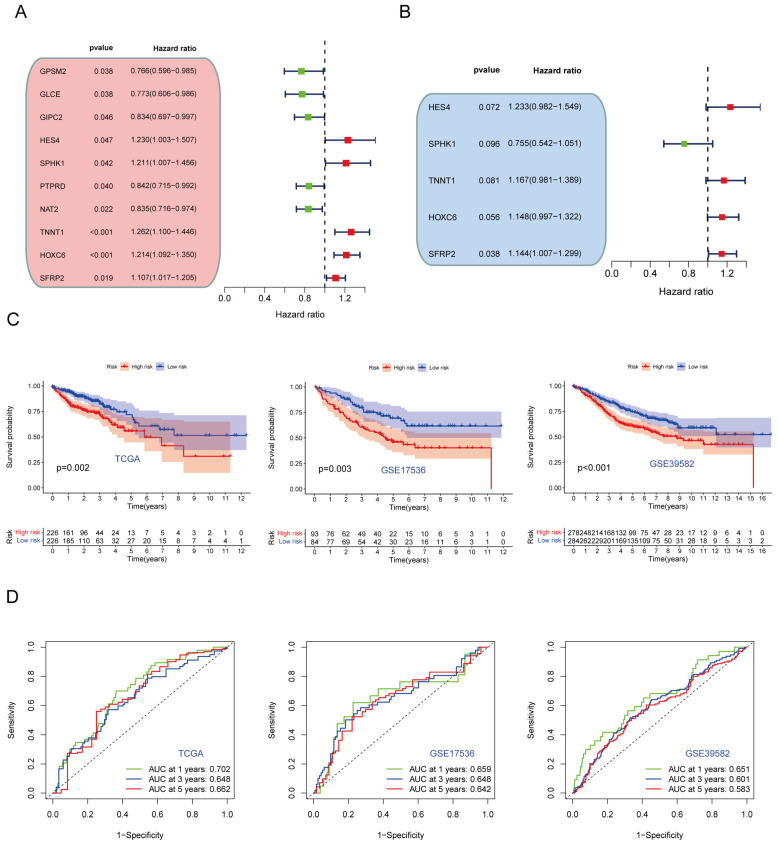
Construction and validation of the prognostic signature. (**A**) Univariate and (**B**) multivariate Cox regression analysis identifies prognosis-related genes. (**C**) Kaplan–Meier survival curves for patients in the high and low CPS-score groups in the training and test sets. (**D**) Receiver operating characteristic curves for 1-, 3-, and 5-year survival in the training and validation sets.

Univariate Cox regression and multivariate Cox regression were performed on 120 DEGs in the training set to identify independent prognostic genes (Figure 4A). Multivariate Cox regression analysis ultimately identified five pivotal genes used to establish the prognostic signature, including hes family bHLH transcription factor 4 (*HES4*), sphingosine kinase 1 (*SPHK1*), troponin T1, slow skeletal type (*TNNT1*), homeobox C6 (*HOXC6*) and secreted frizzled-related protein 2 (*SFRP2*) (Figure 4B). The CPS score is formulated as a function of multiplying the values of the expression of the five model-built genes with their coordinating coefficients, and then summing the output as follows: CPS-score = (0.2096 × HES4) + (−0.2807 × SPHK1) + (0.1546 × TNNT1) + (0.1380 × HOXC6) + (0.1342 × SFRP2). We divided CRC patients into high and low CPS-score groups based on the median cut-off value. Kaplan–Meier survival curves showed that patients in the low CPS-score group had better OS than the high CPS-score group in both the training and test cohorts (TCGA: *p* = 0.02, GSE17536: *p* = 0.003, GSE39582: *p* < 0.001, Figure 4C). The ROC curves showed that the signature effectively predicted 1, 3, and 5-year survival in CRC patients (Figure 4D).

### 3.5. Prognostic Value and Clinical Relevance of the CPS-Score

**Figure 5:**
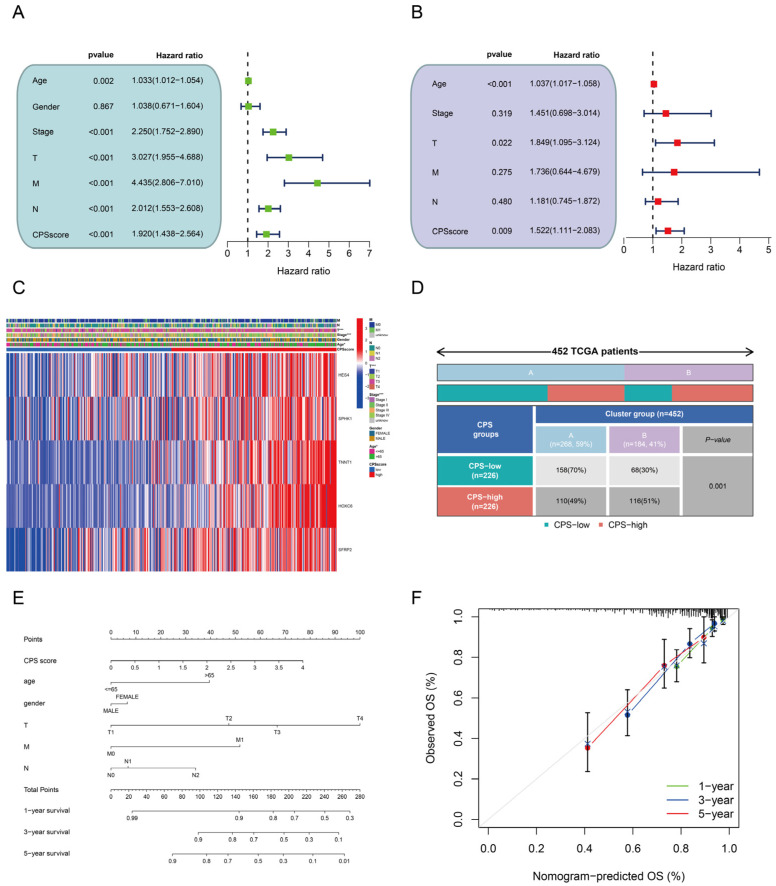
Clinical characteristics analysis of the CPS score. (**A**) Univariate and (**B**) multivariate Cox analysis based on CPS score and clinical covariates. (**C**) The heatmap shows the clinicopathological characteristics and the expression levels of five model genes. (**D**) Correlation between high and low CPS-score groups and cuproptosis subtypes. (**E**) A nomogram that predicts overall survival at 1-, 3-, and 5 years in patients with colorectal cancer. (**F**) Calibration curve of the nomogram. * *p* < 0.05; *** *p* < 0.001.

In the univariate Cox regression analysis, the hazard ratio and 95% confidence range of the CPS score were 1.920 and 1.438–2.564, respectively (Figure 5A); in the multivariate Cox regression analysis, the hazard ratio and 95% confidence range of the CPS score were 1.522 and 1.111–2.083, respectively (Figure 5B). Additionally, we identified two independent clinical prognostic variables, age (1.037, 1.017–1.058, *p* < 0.001) and T-stage (1.849, 1.095–3.124, *p* = 0.022) (Figure 5B). Subsequently, clinical correlations showed that patient age (*p* = 0.044), tumor stage (*p* < 0.001), T-stage (*p* < 0.001), and CPS cluster (*p* < 0.001) were all significantly associated with the CPS score (Figure 5C). The majority of patients in CPS cluster-B had higher CPS scores (Figure 5D), further confirming the accuracy of the CPS score. In addition, patients with stages II and IV, age > 65, and high depth of tumor infiltration (T3-4) also had a higher CPS score (Supplementary Figure S2A). We further proceeded with K-M analysis stratified by various clinical traits and found that CRC patients in the high CPS-score group had worse outcomes in the subgroups comprising ≤ 65 years, >65 years, female, M0, M1, N1–2, III–IV, and T3–T4 stages, while the T1–2, N0, stage I–II, and male subgroup differences were not statistically significant (Supplementary Figure S2B). A nomogram was also constructed to predict 1-, 3-, and 5-year survival rates for colorectal cancer patients (Figure 5E). The calibration curve showed that the nomogram had a good predictive value (Figure 5F).

### 3.6. Immune Microenvironmental Profiling of the CPS Score

**Figure 6:**
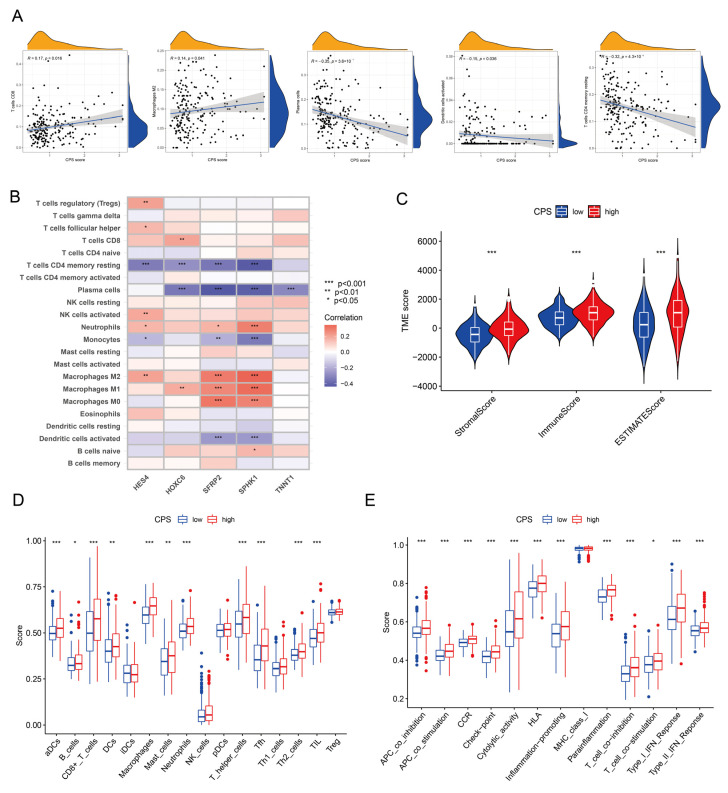
Extended application of CPS score in the immune landscape. (**A**) Correlation analysis between CPS score and immune infiltrating cells. (**B**) Correlation between model-built genes and immune cells. (**C**) ESTIMATE analysis of diverse CPS-score subgroups. (**D**) ssGSEA analysis of diverse CPS-score subgroups for immune infiltrating cells and (**E**) immune-related function. ssGSEA, single-sample gene set enrichment analysis. * *p* < 0.05; ** *p* < 0.01; *** *p* < 0.001.

The CIBERSORT algorithm further analyzed the expression levels of 22 tumor-infiltrating immune subpopulations in CRC and estimated their relevance to the CPS score. The Pearson correlation analysis revealed seven TICs significantly correlated with the CPS score. CD8 T cells (r = 0.17, *p* < 0.016) and M2 macrophages (r = 0.14, *p* < 0.041) were positively correlated with the CPS score, while plasma cells (r = −0.35, *p* < 0.001), activated dendritic cells (r = −0.15, *p* = 0.036), and CD4 memory resting T cells (r = −0.32, *p* < 0.001) were negatively correlated with the CPS score (Figure 6A). The five pivotal genes were differentially correlated with immune cells (Figure 6B). Among them, *HES4* had the highest negative association with resting memory CD4 T cells (*p* < 0.001) and the highest positive association with T cells regulatory (Tregs) (*p* < 0.01); *SFRP2* had the highest positive association with M0 macrophages (*p* < 0.001) and the highest negative association with plasma cells (*p* < 0.001); *SPHK1* had the highest positive association with M2 macrophages (*p* < 0.001) and the highest negative association with CD4 memory resting T cells (*p* < 0.001); *HOXC6* had the highest positive association with CD8 T cells (*p* < 0.01) and the highest negative association with plasma cells; *TNNT1* had the highest negative association with plasma cells (*p* < 0.001). According to the ESTIMATE analysis, CRC patients in the high CPS-score group had higher immune scores, stromal scores, and ESTIMATE scores (*p* = 0.001, Figure 6C). Utilizing ssGSEA, we observed that the level of immune cell infiltration differed dramatically between the distinct CPS-score groups, with 11 of the 16 immune cell species present showing considerable enrichment in high CPS-score groups (Figure 6D). The immune-related function scores were notably enriched in the high CPS-score group, apart from MHC class I which was not significantly different (Figure 6E).

### 3.7. Analysis of Immunotherapy Response Indicators

**Figure 7:**
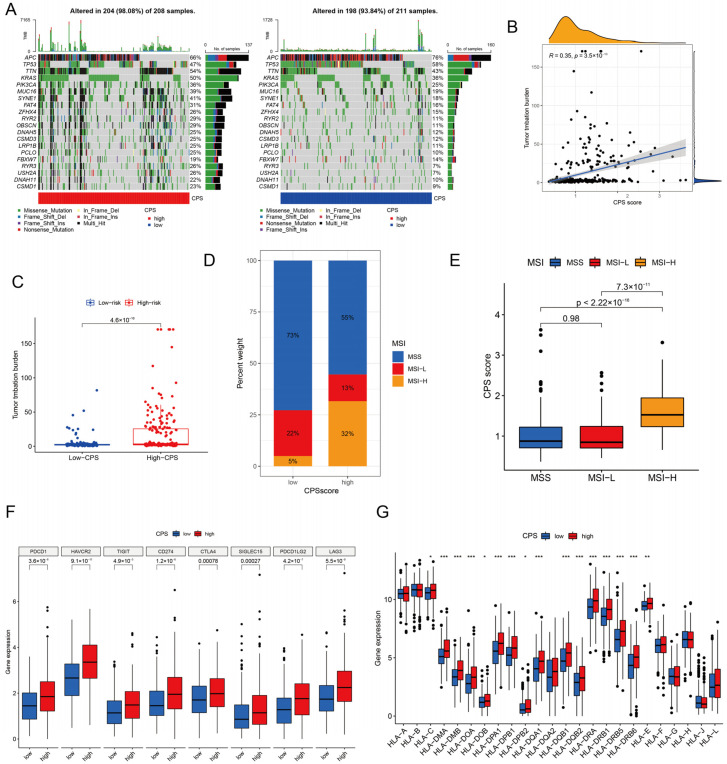
Immunotherapy response analysis of CPS score. (**A**) Waterfall plot of tumor somatic mutations in diverse CPS-score subgroups. (**B**) Correlation between CPS score and TMB. (**C**) Relative distribution of TMB between the high and low CPS-score groups. (**D**) MSI characteristics of the diverse CPS-score subgroups. (**E**) Differences in CPS score between MSS, MSI-L, and MSI-H. (**F**) Differences in ICB expression between diverse CPS-score subgroups. (**G**) Differences in expression of HLA-related genes between high and low CPS-score groups. TMB, tumor mutation burden; MSI, microsatellite instability; MSS, microsatellite stability; MSI-L, low-frequency MSI; MSI-H, high-frequency MSI; ICB, immune checkpoint blockade; HLA, human leukocyte antigen. * *p* < 0.05; ** *p* < 0.01; *** *p* < 0.001.

The differences in the distribution of somatic mutations between the high CPS-score and low CPS-score groups were further examined. The results revealed that the high CPS-score group (98.08%) exhibited a more comprehensive range of somatic mutations than the low CPS-score group (93.84%) (Figure 7A). We also observed that the CPS score and TMB showed a remarkable positive correlation (r = 0.35, *p* < 0.001, Figure 7B). Quantitative analysis also confirmed that the high CPS-score group had higher TMB values than the low CPS-score group (*p* < 0.001, Figure 7C). More importantly, we also explored the MSI characteristics of the high and low CPS-score groups, and the results showed that patients in the low CPS-score group had higher MSI-H ratios (Figure 7D,E). Subsequently, we checked the expression of eight ICBs, including *CD274*, *CTLA4*, *HAVCR2*, *TIGIT*, *LAG3*, *PDCD1*, *PDCD1LG2*, and *SIGLEC15*, and the results showed that all eight ICBs were highly expressed in the high CPS-score subgroup (Figure 7F). Also investigated was the relationship between CPS score and HLA-related gene expression. The results showed that the major HLA I (including HLA-C and HLA-E) and HLA II-related genes (including *HLA-DMA*, *HLA-DMB*, *HLA-DOA*, *HLA-DOB*, *HLA-DPA1*, *HLA-DPB1*, *HLA-DPB2*, *HLA-DQA1*, *HLA-DQB1*, *HLA-DQB2*, *HLA-DRA*, *HLA-DRB1*, *HLA-DRB5*, *HLA-DRB6*, and *HLA-E*) were significantly differentially expressed, and all positively correlated with the CPS score (Figure 7G). 

### 3.8. Single-Cell Transcriptomic Analysis

**Figure 8:**
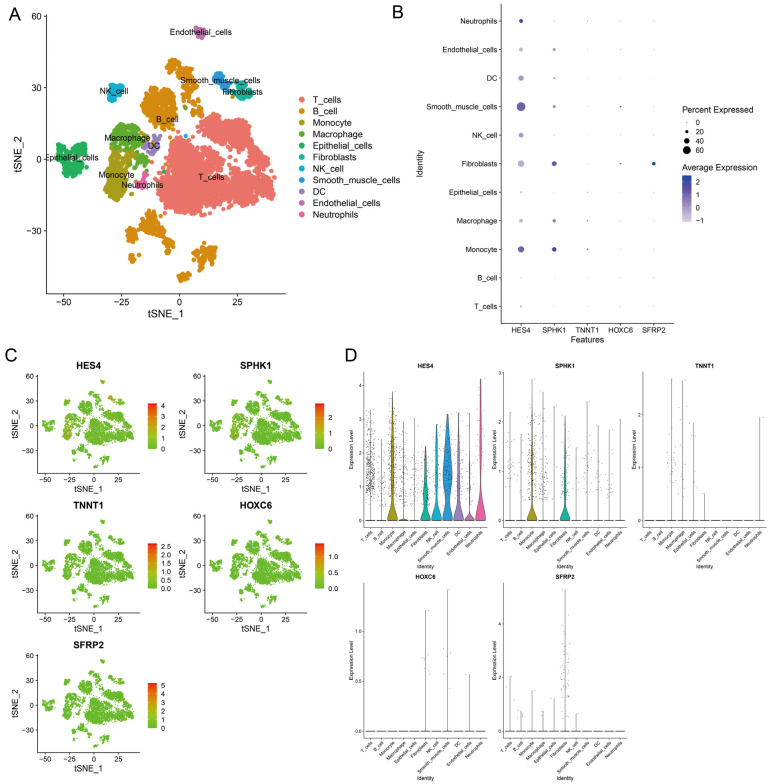
CRC single-cell profiles. (**A**) tSNE plots for 11 cell types. (**B**) The bubble plots show the expression levels of model-built genes in all cell types. (**C**) The tSNE plots show the expression levels of model-built genes in all cell types. (**D**) The violin plots show the expression levels of model-built genes in all cell types.

After applying stringent quality control to GSE18871, sequencing depth was positively correlated with mRNA number but not significantly with mitochondrial gene sequence (Supplementary Figure S3A). PCA analysis showed a good removal of batch effects (Supplementary Figure S3B) and determined the optimal number of major components (Supplementary Figure S3C). Then, tSNE analysis was performed for downscaling and visualization, and cells were clustered into 21 major clusters (Supplementary Figure S3D). These clusters were labeled as T cell, B cell, monocyte, macrophage, epithelial cell, fibroblast, NK cell, smooth muscle cell, dendritic cell, endothelial cell, dendritic cell, and neutrophil based on typical markers and genetic profiles in colorectal cancer tissues (Figure 8A). We examined the expression profiles of five model genes in 11 cell types. Heatmap visualized the expression of each gene in the 11 cell types (Figure 8B). The distribution of model genes in different cell types was visualized by tSNE plots (Figure 8C). Violin plot showed that *HES4* was significantly expressed in monocytes, macrophages, fibroblasts, NK cells, smooth muscle cells, dendritic cells, endothelial cells, and neutrophils, and *SPHK1* was significantly expressed in monocytes and fibroblasts (Figure 8D).

### 3.9. Drug Response Analysis

**Figure 9:**
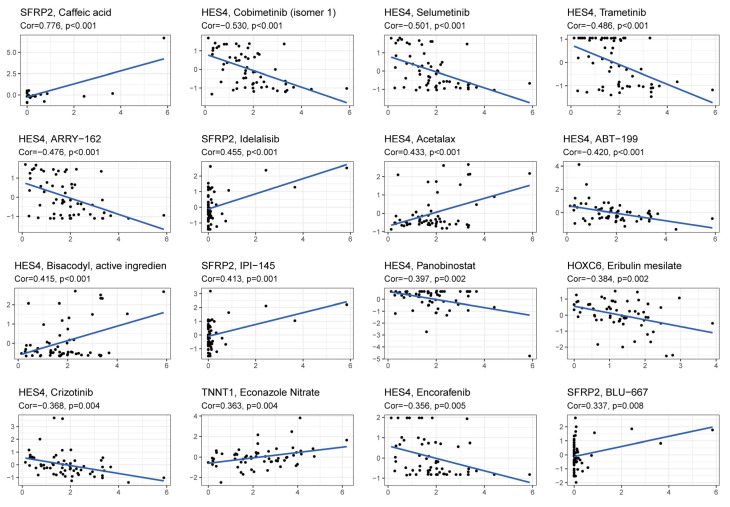
Correlation analysis of model genes and drug sensitivity.

In addition, we analyzed the relationship between five key genes and drug sensitivity using the CellMiner database, and all drugs significantly associated with hub gene expression are listed in Supplementary Table S6. The 16 drugs with the lowest *p*-values in the correlation analysis are illustrated in Figure 9. Among them, *HES4* was negatively associated with sensitivity to cobimetinib (isomer 1), selumetinib, trametinib, ARRY-162 (binimetinib), ABT-199 (venetoclax), panobinostat, crizotinib, encorafenib, and with acetalax, bisacodyl, the active ingredients were significantly positively correlated. *SFRP2* expression was positively correlated with sensitivity to caffeic acid, idelalisib, IPI-145 (duvelisib), and BLU-667 (pralsetinib). *HOXC6* was negatively correlated with eribulin mesylate sensitivity. *TNNT1* was positively correlated with econazole nitrate sensitivity.

### 3.10. Verification of Hub Gene Expression Levels

**Figure 10:**
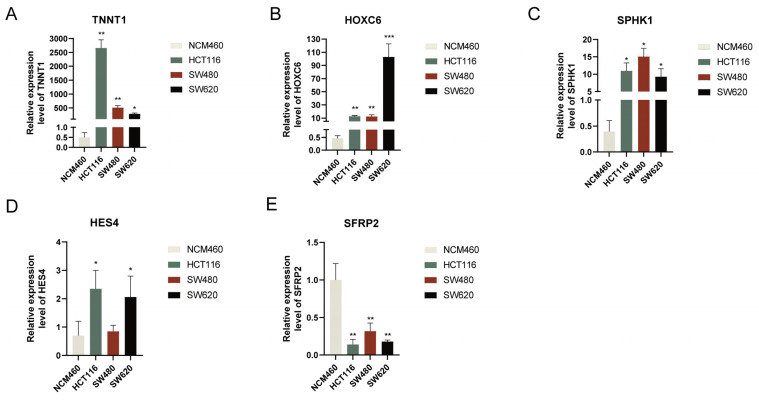
Validation of mRNA expression levels of signature genes in colorectal cancer cell lines. The expression of (**A**) TNNT1, (**B**) HOXC6, (**C**) SPHK1, (**D**) HES4, and (**E**) SFRP2 were detected by RT-qPCR in CRC cell lines. * *p* < 0.05; ** *p* < 0.01; *** *p* < 0.001.

We performed an RT-qPCR analysis of NCM460 and HCT116, SW480, and SW620 cells. The results revealed that *TNNT1*, *HOXC6*, and *SPHK1* were expressed markedly higher in CRC cell lines (HCT116, SW480, and SW620) than in normal cell lines, *HES4* was highly expressed in HCT116 and SW620 cell lines, and *SFRP2* was significantly lower in colorectal cancer cell lines (HCT116, SW480, and SW620) (Figure 10). Taken together, the aberrant expression of these five genes was further validated in CRC cell lines.

## 4. Discussion

Despite recent advances in surgical techniques, conventional chemotherapy, radiotherapy, and neoadjuvant therapy, there has not been sufficient progress in survival rates for advanced CRC [26,27]. Due to the high degree of heterogeneity and sophistication of CRC, traditional histological and anatomical classifications have significant limitations in guiding antitumor therapy [28]. Therefore, the accurate identification of molecular subtypes and the establishment of genetic signatures of CRC are crucial to guide the personalized treatment of CRC patients. It is thought that cuproptosis may regulate cancer cell death by targeting mitochondrial respiration as a new form of copper-dependent death [14]. Elesclomol has recently been reported to exhibit anticancer activity via the induction of cuproptosis, and its virulence against cancer cells is strongly determined by the transportation of copper ions in the extracellular environment [29]. There was also a significant inhibitory effect of elesclomol on several drug-resistant cancer cells that was associated with enhanced mitochondrial metabolism in these cells. It was found that elesclomol could induce copper-dependent ferroptosis in CRC by stimulating the breakdown of copper-transporting ATPase 1, thereby delaying cancer progression [30]. Researchers have confirmed that cuproptosis-related molecular signatures affect the prognosis and effectiveness of anticancer treatments among patients with a wide range of cancers [31,32,33]. For instance, Ma et al. built a cuproptosis-associated long noncoding RNA prognostic model in lung adenocarcinoma, which could be a satisfactory predictor of prognosis and immunotherapy in lung cancer patients [34]. Liu et al. distinguished kidney cancer into “cold tumors” and “hot tumors” based on cuproptosis regulators and formulated a double gene model for forecasting antitumor treatment response in kidney cancer [35]. *LIPT1*, a cuproptosis-related gene, was found to accelerate the growth and metastasis of hepatocellular carcinoma and is potentially a promising therapeutic target for hepatocellular carcinoma [36]. Therefore, the current study investigated the potentially crucial function of the cuproptosis modulator that could enhance the treatment of CRC and improve its prognosis.

We first systematically analyzed the expression of 19 CRs in CRC and normal tissues and identified 14 DECRs. Next, we assessed the genetic variation in CRs and found that 81 of 452 patients had mutations with a mutation frequency range of 1–5%, with the most frequent mutations being in *NLRP3* and *ATP7A* (5%). Significant copy number amplification of *ATP7B* and *NLRP3* was also observed, while *DBT* and *PDHB* had significant copy number deletions. We then consolidated the transcriptome data of 1191 colorectal cancers and defined two distinct cuproptosis subtypes in CRC. A markedly different survival time and TME cell infiltration characteristics were observed for these two molecular subtypes. Further studies of cuproptosis-associated DEGs showed that they can also be divided into two genetic subtypes in CRC and are significantly associated with different survival outcomes. 

To guide the treatment strategy for individual CRC patients more accurately, we constructed a cuproptosis prognostic model resulting in the recruitment of five genes (*HES4*, *SPHK1*, *TNNT1*, *HOXC6*, and *SFRP2*). RT-qPCR results showed that *HES4*, *TNNT1*, *HOXC6*, and *SPHK1* were highly expressed in CRC cell lines, while *SFRP2* was lowly expressed in CRC cell lines. *TNNT1* is a subunit of troponin that has been found to be upregulated in a variety of tumor tissues, including breast, colorectal and endometrial cancers [37,38,39]. Chen et al. demonstrated that *TNNT1* is negatively regulated by miR-873 and is an oncogene associated with poor patient prognosis [40]. *HOXC6* is a member of a family of homozygous cassette genes encoding highly conserved transcription factors that play a critical role in a variety of tumors [41]. Ji et al. demonstrated through tissue microarrays that *HOXC6* was significantly highly expressed in colorectal cancer tissues and was associated with a poorer prognosis, higher tumor stage, and lymph node metastasis [42]. Additionally, *HOXC6* can promote CRC progression by regulating autophagy and the mTOR pathway. *SPHK1* is a crucial member of the sphingosine kinase family participating in the regulation of numerous cancer-related bio-processes, such as cell proliferation, invasion, and angiogenesis, and is associated with prognosis in a wide range of cancers [43,44]. Liu et al. showed that *SPHK1* could promote colorectal cancer through the induction of FAK/AKT/MMPs axis-mediated epithelial-mesenchymal transition to promote metastasis in colorectal cancer [45]. Another study also found that SPHK1-driven autophagy may promote CRC metastasis through the induction of paxillin expression and phosphorylation [46]. Secretory frizzled-related protein (SFRP) is a critical participant in Wnt signaling and tumor angiogenesis and is usually differentially expressed in the tumor microenvironment [47]. *SFRP2* methylation in stool has been reported to be a promising biological marker for early CRC detection [48]. Bai et al. found that the overexpression of *SFRP2* inhibited CRC cell proliferation and promoted apoptosis [49], while several other studies have found a significantly higher expression of *SFRP2* in metastatic tumors (including osteosarcoma, breast cancer, and malignant melanoma) [50,51,52]. The Notch signaling pathway is an essential regulator of cell proliferation, apoptosis, and differentiation, and plays a crucial role in the progression of many tumors [53,54]. As a downstream target gene of Notch signaling, *HES4* has been reported to act as a prognostic biomarker for various tumors, such as breast cancer and osteosarcoma [53,54,55]. These findings suggest a critical role for our model-built genes in the progression of various cancers.

The composition and degree of infiltration of immune cells in TME perform a critical factor in tumor progression, prognosis, and treatment. Macrophages at chronic inflammatory sites suitable for tumor progression have been reported to exhibit an M1 phenotype. However, as tumors progress, M1 macrophages polarize and change their characteristics to an M2-like phenotype [56]. M2 phenotype macrophages can impede the immune activation of T cells by recruiting Tregs and secreting anti-inflammatory cytokines to facilitate tumor immune escape [57]. Regulatory T cells (Tregs) protect against autoimmunity; the infiltration of Tregs accumulation in tumors, however, can suppress the expansion of antitumor effector T cells and facilitate the formulation of an immunosuppressive microenvironment [58]. In this study, the infiltration rate of M2 macrophages was significantly higher in patients with high CPS scores, predicting a worse prognosis for these patients. Correlation analysis showed that *HES4* expression was positively associated with the richness of Tregs infiltration, and *SPHK1* expression was positively associated with the level of M2 macrophages. The validation of expression levels based on single-cell sequencing datasets also showed that *HES4* was significantly expressed in a variety of tumor-infiltrating immune cells (such as dendritic cells, monocytes, NK cells, macrophages, and neutrophils) and stromal cells (fibroblasts, smooth muscle cells, and endothelial cells). *SPHK1* was expressed at high levels in monocytes and fibroblasts. Numerous studies have shown that stromal cell populations, particularly tumor-associated fibroblasts (CAFs), a major component of the tumor stroma, promote CRC invasion and metastasis [59]. The above results suggest that the CPS score plays an important role in the involvement of CRC progression and the formation of an immunosuppressive microenvironment.

In recent years, immune checkpoint blockade (ICB) therapy has become increasingly popular and has made significant progress in CRC. Several studies have shown that immunotherapy can significantly improve overall survival (OS) and progression-free survival (PFS) in patients with progressive CRC [60,61]. However, it is frustrating that only a tiny proportion of CRC patients with MSI-H/d-MMR can benefit from immunotherapy [62]. It is well known that immunogenic tumors respond better to treatment with ICB than nonimmunogenic tumors and that the intensity of immune checkpoint inhibitors and MHC type I expression determines the effectiveness of immunotherapy [63]. Here, the analysis of immune checkpoint expression profiles showed that all eight immune checkpoint genes and major HLA I and II-associated genes had higher expression profiling levels in the high CPS-score group of patients, predicting that the high CPS-score group may have a better response to immunotherapy. Recent studies have shown that TMB is an emerging predictive biomarker for anti-PD 1/anti-PD L1 therapy and other immunotherapeutic agents [64]. In various cancers, a high TMB has been shown to be associated with better OS in patients receiving immunotherapy [65]. CRC patients with MSI-H/d-MMR typically have an increased TMB compared to MSS/pMMR patients and have a better therapeutic response to ICB due to continuous antigen renewal by unrepaired misreplicating DNA, which allows for greater infiltration of TILs [66]. Our data show a significant positive correlation between CPS score and TMB and MSI-H, suggesting that patients in the high CPS-score group have higher immunogenicity, which further demonstrates that patients in the high CPS-score group are potentially a high-beneficiary category for ICB treatment. Our findings provide a basis and framework for a better understanding of patient response to immunotherapy and a better tool to guide more personalized and effective immunotherapy strategies.

Multidrug resistance is an underlying cause of chemotherapeutic failure in colorectal cancer [67]. Conventional chemotherapeutic agents are inevitably limited in patients with metastatic colorectal cancer due to acquired or intrinsic drug resistance [68]. Therefore, we investigated the connection between model genes and anticancer drug sensitivity to provide new insights for exploring tumor therapy and avoiding tumor drug resistance. The majority of model-built genes were found to be associated negatively with anticancer drug sensitivity. For example, *HES4* was negatively related to the sensitivity of cobimetinib, selumetinib, trametinib, binimetinib, crizotinib, and encorafenib. Cobimetinib, selumetinib, trametinib, and binimetinib are all mitogen-activated protein kinase (MEK) inhibitors and are the first MAPK pathway inhibitors to enter clinical trials [69]. Several clinical trials have reported the antitumor activity of MEK inhibitors in CRC patients with BRAF or KRAS mutations [70,71]. Gong et al. found that cobimetinib not only inhibited CRC cell proliferation but also induced G1 phase arrest and apoptosis in cancer cells; in addition, cobimetinib appeared to enhance the efficacy of 5-Fluorouracil (5-FU) by reducing TYMS expression [72]. Zhang et al. found a therapeutic sequence-dependent synergistic effect of selumetinib and 5-FU in KRAS or BRAF mutant CRC models [73]. Wang et al. found that MDM4/MDM2 double knockdown combined with trametinib enhanced P53 activation, thereby exerting antitumor effects through the induction of the G1 blockade and apoptosis in wild-type TP53 colorectal cancers with aberrant KRAS signaling [74]. Crizotinib was initially defined as a selective ATP-competitive MET inhibitor and was later found to inhibit several related kinases, including allogeneic lymphoma kinase (ALK) and c-Ros oncogene 1, receptor tyrosine kinase (ROS1) [75]. Crizotinib has been proven to have a significant response and clinical benefit in patients with ROS1 genomic rearrangements or ALK fusion-positive colorectal cancer [76,77]. More recently, encorafenib (BRAF kinase inhibitor) combined with cetuximab has been reported for second or third-line treatment regimens in BRAF V600E-mutated CRC [78,79]. In a clinical trial in patients with BRAF V600E mutant metastatic CRC, encorafenib plus cetuximab was associated with significantly prolonged survival and remission rates compared with standard therapy [80]. The above results indicate that the CPS score may help provide patients with an appropriate chemotherapy strategy.

There are also some limitations to our study. First, our data were largely derived from TCGA and GEO databases and no external validation was conducted. Moreover, we have verified the expression profiling at the cellular level, and further cell functional assays and in vivo animal model construction may better reveal the particular mechanisms and effects of pivotal genes in colorectal cancer. 

## 5. Conclusions

In summary, we constructed and validated a five-gene prognostic model based on CRs which can effectively predict the OS of CRC patients and serve an essential part in the future precision therapy of CRC patients.

## Data Availability

The raw RNA-seq data generated in this study are available in the TCGA and GEO public databases. All remaining data are available in the article, in the Supplementary Information File, or from the corresponding author upon reasonable request.

## Supplementary Materials

The following supporting information can be downloaded at: https://www.mdpi.com/article/10.3390/cancers15020387/s1, Figure S1: Identification of gene subgroups based on DEGs between molecular subtypes; Figure S2: Clinicopathologic characteristics and survival subgroup analysis based on the CPS-score; Figure S3: Single-cell RNA sequencing analysis of CRC tissues. Table S1: The clinical characteristics of the TCGA cohort, GSE17536 and GSE39582 cohort; Table S2: 19 cuproptosis-related genes; Table S3: qPCR primers; Table S4: GSVA enrichment analysis of cuproptosis-related molecular subtypes; Table S5: Differentially expressed genes between cuproptosis-related molecular subtypes; Table S6: Correlation analysis between model gene expression and drug sensitivity.
