# Supplementary material for: Multiomics Analysis Reveals Cuproptosis-Related Signature for Evaluating Prognosis and Immunotherapy Efficacy in Colorectal Cancer
Source: Cancers (Basel). 2023 Jan 6;15(2):387. doi: 10.3390/cancers15020387 (PMC9856392; doi:10.3390/cancers15020387)
Supplement: Supplementary file 1 [file cancers-15-00387-s001.zip › Supplementary figures.pdf]

Supplementary Figures

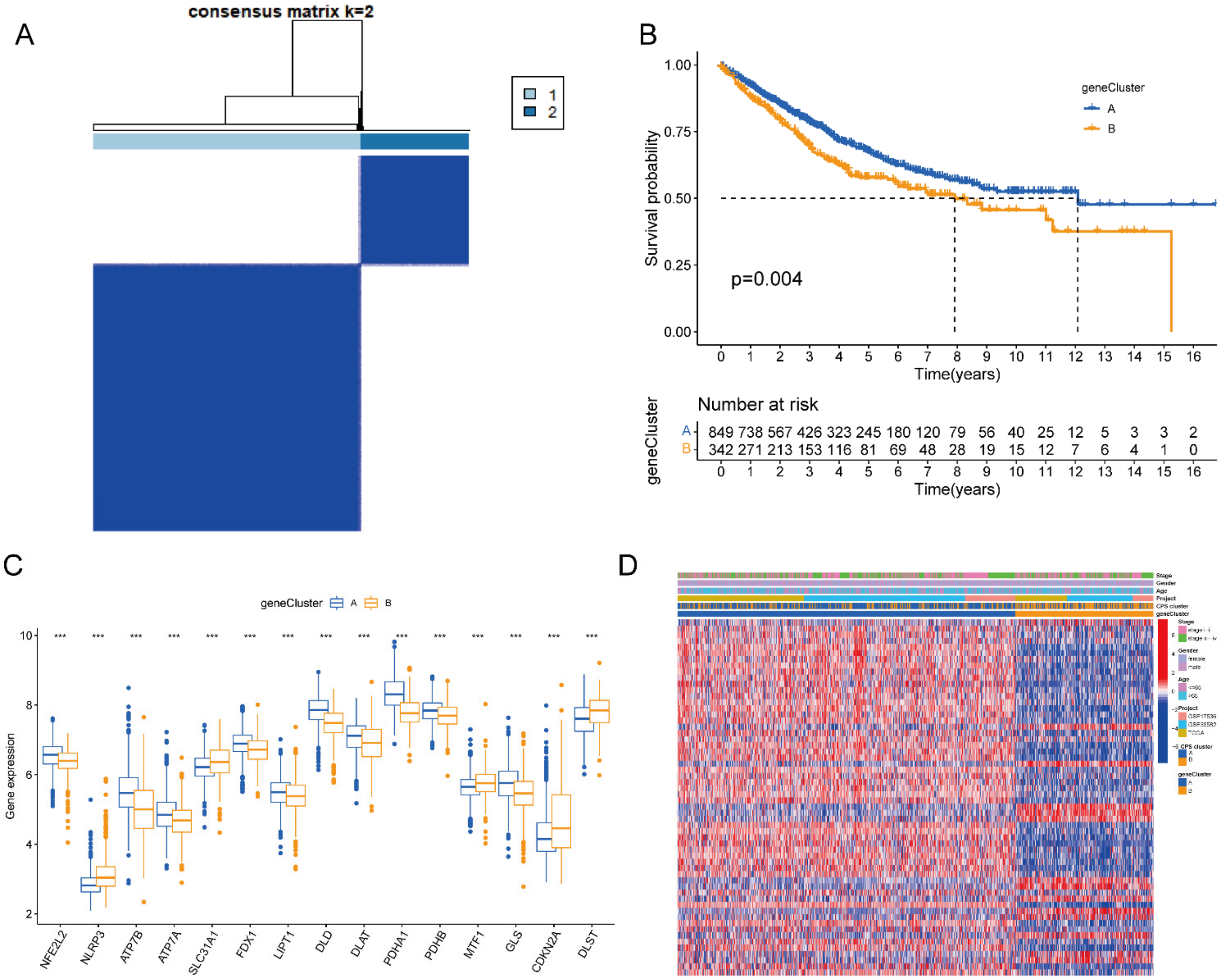

Supplementary Figure S1. Identification of gene subgroups based on DEGs between molecular subtypes. (A) Consensus matrix at group number (k) = 2. (B) Kaplan-Meier survival analysis between two different gene subgroups. (C) Differential expression of DECRGs in the two gene subgroups. (D) Heatmap of clinicopathological characteristics and expression of DEGs between gene subgroups. DEG, differentially expressed gene; DECRG, differentially expressed cuproposis-associated gene. *** $p < 0.001$.

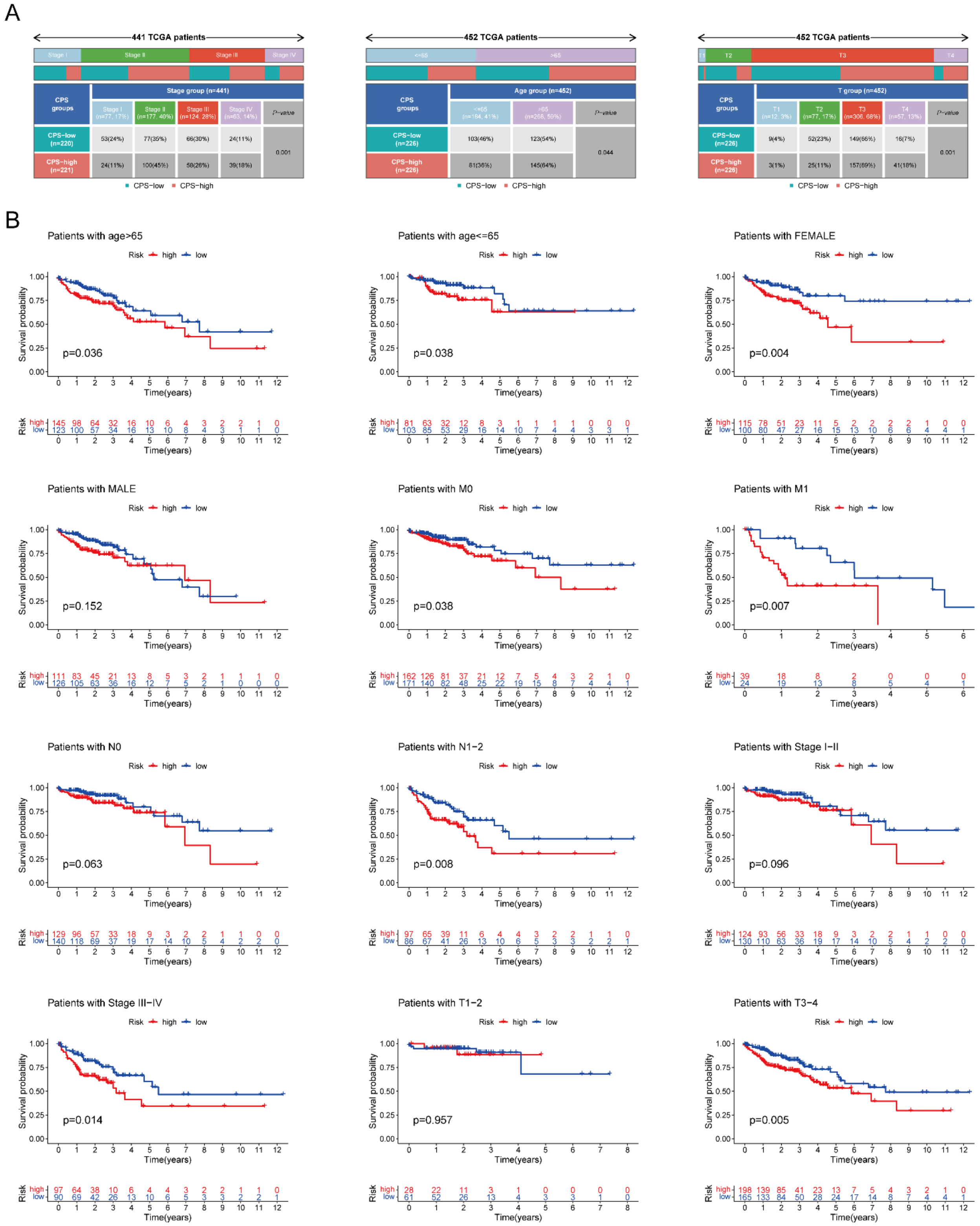

Supplementary Figure S2. Clinicopathologic characteristics and survival subgroup analysis based on the CPS-score. (A)Correlation between tumor stage, age, and depth of tumor infiltration (T-stage) and CPS-score. (B)Kaplan-Meier survival analysis of different subgroups of clinical characteristics between the high CPS-score and low CPS-score groups.

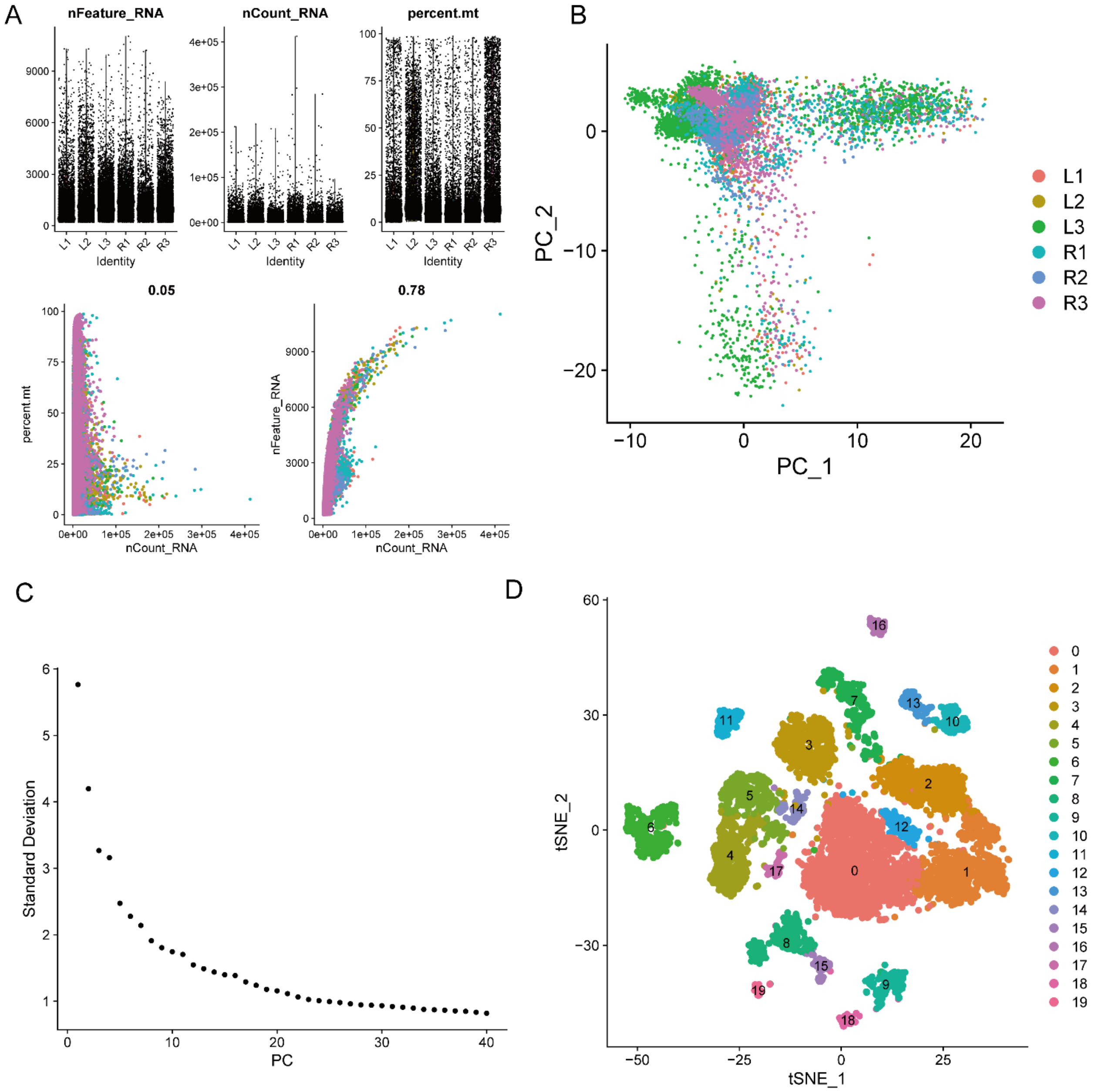

Supplementary Figure S3. Single-cell RNA sequencing analysis of CRC tissues. (A) Post-quality control filtering of each sequenced cell with violin plots showing its RNA feature number (nFeature RNA), absolute UMI count (nCount RNA), and mitochondrial content (percent. mt) and showing their correlation. (B) PCA of single-cell data from 6 tissues. (C) Elbow Plot of PCA. (D) Cells were clustered into 19 types by the tSNE dimensionality reduction algorithm, with each color representing the annotated phenotype of each cluster. CRC, colorectal cancer; PCA, principal component analysis.
